# Influence of *Bifidobacterium breve* on the Glycaemic Control, Lipid Profile and Microbiome of Type 2 Diabetic Subjects: A Preliminary Randomized Clinical Trial

**DOI:** 10.3390/ph16050695

**Published:** 2023-05-04

**Authors:** Chaiyavat Chaiyasut, Bhagavathi Sundaram Sivamaruthi, Narissara Lailerd, Sasithorn Sirilun, Subramanian Thangaleela, Suchanat Khongtan, Muruganantham Bharathi, Periyanaina Kesika, Manee Saelee, Thiwanya Choeisoongnern, Pranom Fukngoen, Sartjin Peerajan, Phakkharawat Sittiprapaporn

**Affiliations:** 1 Innovation Center for Holistic Health, Nutraceuticals, and Cosmeceuticals, Faculty of Pharmacy, Chiang Mai University, Chiang Mai 50200, Thailand; sivamaruthi.b@cmu.ac.th (B.S.S.);; 2 Office of Research Administration, Chiang Mai University, Chiang Mai 50200, Thailand; 3 Department of Physiology, Faculty of Medicine, Chiang Mai University, Chiang Mai 50200, Thailand; 4 Neuropsychological Research Laboratory, Neuroscience Research Center, School of Anti-Aging and Regenerative Medicine, Mae Fah Luang University, Bangkok 10110, Thailandwichian.sit@mfu.ac.th (P.S.); 5 Health Innovation Institute, Chiang Mai 50200, Thailand

**Keywords:** diabetes mellitus, *Bifidobacterium breve*, probiotics, microbiota, lipid profile

## Abstract

Type 2 diabetes mellitus (T2DM) is one of the most highly prevalent metabolic disorders worldwide. Uncontrolled T2DM can lead to other health threats such as cardiac arrest, lower-limb amputation, blindness, stroke, impaired kidney function, and microvascular and macrovascular complications. Many studies have demonstrated the association between gut microbiota and diabetes development and probiotic supplementation in improving glycemic properties in T2DM. The study aimed to evaluate the influence of *Bifidobacterium breve* supplementation on glycemic control, lipid profile, and microbiome of T2DM subjects. Forty participants were randomly divided into two groups, and they received probiotics (50 × 10^9^ CFU/day) or placebo interventions (corn starch; 10 mg/day) for 12 weeks. The changes in the blood-urea nitrogen (BUN), aspartate aminotransferase (AST), alanine transaminase (ALT), alkaline phosphatase (ALP), fasting blood sugar (FBS), glycated hemoglobin (HbA1c), total cholesterol (TC), triglycerides (TG), high-density lipoprotein (HDL), low-density lipoprotein (LDL), creatinine levels, and other factors such as body-mass index, visceral fat, body fat, and body weight were assessed at baseline and after 12 weeks. *B. breve* supplementation significantly reduced BUN, creatinine, LDL, TG, and HbA1c levels compared to the placebo group. Significant changes were observed in the microbiome of the probiotic-treated group compared to the placebo group. Firmicutes and proteobacteria were predominant in the placebo and probiotic-treated groups. Genera *Streptococcus*, *Butyricicoccus*, and species *Eubacterium hallii* were significantly reduced in the probiotic-treated group compared to the placebo. Overall results suggested that *B. breve* supplementation could prevent worsening of representative clinical parameters in T2DM subjects. The current study has limitations, including fewer subjects, a single probiotic strain, and fewer metagenomic samples for microbiome analysis. Therefore, the results of the current study require further validation using more experimental subjects.

## 1. Introduction

Type 2 diabetes mellitus (T2DM) is a common complex metabolic disorder that is highly prevalent worldwide. This systemic disorder challenges host homeostasis, impairs insulin efficacy, disturbs carbohydrate and lipid metabolism, and creates gut dysbiosis [1]. As per the statistics released by the International Diabetes Federation (IDF), T2DM in adults could rise by 9.9% by 2045, with 693 million people with T2DM [2]. The risk factors associated with T2DM include obesity, low physical activity, and age. T2DM is more frequent in people with hypertension and dyslipidemia [3]. Genetics, a high-energy diet, and physical inactivity may increase the risk of T2DM [4]. In addition to lipid imbalances, the increased level of free fatty acids contributes to T2DM-related insulin resistance [5].

Gut microbiota greatly impact glucose tolerance, insulin resistance, and dyslipidemia. Gut dysbiosis contributes to T2DM development [6]. Different factors, including diet, influence gut-microbiota diversity, which may be linked to T2DM, obesity, cardiovascular diseases, and other health complications [7,8,9]. A sequencing study was carried out among healthy and T1DM subjects, and the results showed significant differences in the gut-microbial profile [10]. Gut-microbial dysbiosis due to a reduced number of butyrate-producing commensals may induce gut permeability and allow the release of microbial metabolites, which eventually cause immune activation and the onset of metabolic diseases such as diabetes [11]. Factors such as gut-barrier functions, gut microbiota, and a high-fat diet (HFD) influence T2DM progression by inducing inflammatory pathways, metabolic endotoxemia, and metabolic disorders [12,13].

Probiotics are live microorganisms that, when administered in adequate amounts, confer a health benefit on the host [14]. Probiotics are reported to have several health benefits, including for metabolic disorders, aging-associated complications, and cognitive decline [15,16,17,18,19]. Studying the interrelationship among gut microbiota, high-energy-rich diets, and the influence of probiotics could provide improved strategies to prevent or manage T2DM. Probiotic supplementation may improve the fasting-blood-glucose level, insulin sensitivity, inflammatory and antioxidant systems, gut-microbial composition, and amelioration of gut permeability. Improved gut permeability and reduced endotoxemia were observed in probiotic-supplemented T2DM subjects [20]. Our previous study reported that supplementation with *Lactobacillus paracasei* HII01 (50 × 10^9^ CFU/day for 12 weeks) improved hyperglycemia and certain inflammatory markers by regulating gut microbiota, and enhanced gut permeability and reduced endotoxemia. *L. paracasei* HII01 supplementation also increased the beneficial microbial load and reduced the pathogens’ load in T2DM subjects [21].

The anti-diabetic effects of *Bifidobacterium animalis* (*B. animalis*) 01 were reported in T2DM-induced mice. Oral supplementation of *B. animalis* 01 reduced blood-glucose and glycosylated-hemoglobin (HbA1c) levels with improved lipid profiles, cytokines, and liver-injury markers. *B. animalis* 01 also decreased hepatic-tissue injuries and enhanced antioxidant levels. These results cumulatively revealed the hepatoprotective effects of *B. animalis* 01 in T2DM rats [22]. Administration of *B. adolescentis* 3M10 downregulated lipid metabolism and retinoic-acid-inducible gene-I (RIG-I)-like receptor signaling. Downregulation of RIG-I expression inhibits p38 mitogen-activated protein-kinase and nuclear-factor kappa B (NK-kB) signaling pathways, suppressing pro-inflammatory-factor production [23]. *B. adolescentis* restored gut homeostasis, developed short-chain fatty-acid (SCFA)-producing microbiota, and inhibited inflammation in T2DM mice. *B. adolescentis* can colonize in the adult gut and alleviate T2DM-associated complications. *B. adolescentis* strains reduced serum interleukin-6 (IL-6), tumor-necrosis factor-α (TNF-α), and interferon-γ (IFN-γ) levels in mice treated with a high-fat diet and streptozotocin. *Bifidobacterium*-supplemented mice showed a high concentration of fecal SCFAs (acetic, propionic, and butyric acids) and improved inflammation and hypoglycemia [24].

The objective of the present study was to validate the efficiency of *Bifidobacterium breve* (*B. breve*) supplementation on the glycemic control, lipid profile, and microbiome of T2DM subjects.

## 2. Results

Forty T2DM subjects were enrolled and completed the current clinical trial. The baseline characteristics, including age, body weight, visceral fat, body-mass index (BMI), and other biochemical parameters, showed no significant difference between subjects assigned to the placebo and treatment groups (Table 1).

### 2.1. The Efficacy of B. breve Supplementation on Blood Biochemical Parameters

Table 2 represents the biochemical parameters assessed between the placebo and treatment groups and within the groups (Ppre and Ppost; Tpre and Tpost). After 12 weeks, the placebo group had no significant change in body weight, BMI, visceral fat, BUN, creatinine, AST, ALT, FBS, TC, TG, or LDL. Significant changes were observed in body fat (increased), ALP (decreased), HbA1c (increased), and HDL (increased) in the placebo group compared to the baseline values. Creatinine, ALP, and LDL levels had decreased, and HDL levels had increased significantly after 12 weeks in the treatment group compared to the baseline values. Other parameters were not changed significantly in the treatment group (Table 2).

The changes in baseline and after 12 weeks of study in the placebo and treatment groups are provided (Table 2). Table 3 represents the differences in the studied parameters between the placebo and treatment groups at the end of the study. The body weight, body fat, FBS, and HbA1c values were significantly reduced (*p* < 0.05) in the treatment group compared to the placebo after 12 weeks. Other parameters (BMI, visceral fat, BUN, creatinine, AST, ALT, ALP, TC, TG, HDL, and LDL) were not altered significantly (Table 3).

A Gaussian-regression analysis of the study suggested that *B. breve* supplementation for 12 weeks significantly improved the visceral fat (−2.92 to 0.31; *p* = 0.018), FBS (−42.52 to −3.66; *p* = 0.021), HbA1c (−2.43 to −0.28; *p* = 0.016), TC (−56.61 to −1.17; *p* = 0.042), and LDL (−56.58 to −1.20; *p* = 0.041) (Table 4).

### 2.2. Microbiome Analysis

The total bacterial sequences 570,321 (Ppre), 460,273 (Ppost), 681,505 (Tpre), and 277,693 (Tpost) were read in QIIME 2^TM^. Approximately 55,335 (Ppre), 36,532 (Ppost), 474,814 (Tpre), and 124,354 (Tpost) non-chimeric sequences were recovered after filtering, denoising, and combining the non-chimeric sequences.

#### 2.2.1. Microbial Abundances (Alpha-Diversity)

**Figure 1:**
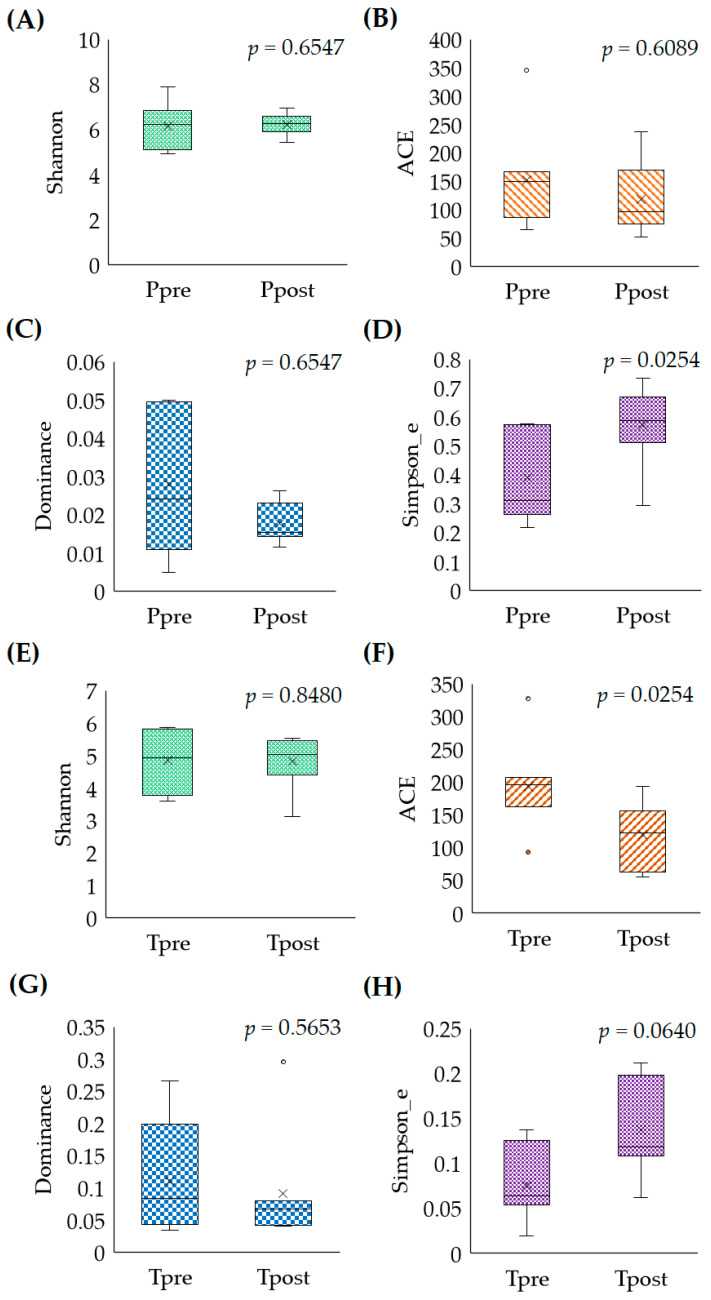
The estimated Shannon index (**A**,**E**), ACE (**B**,**F**), dominance (**C**,**G**), and Simpson evenness (**D**,**H**) of pre-and post-samples of the placebo and treatment groups. The statistical significance for the Shannon index, ACE, and Simpson evenness was *p* ≤ 0.05 and dominance was *p* ≥ 0.05. Ppre: placebo at baseline; Ppost: placebo at week 12; Tpre: treatment at baseline; Tpost: treatment at week 12.

The diversity richness of the fecal samples was estimated using the Shannon index. The Shannon index revealed that the microbial-diversity richness was slightly increased in the Ppost samples with a median value (M) of 6.239 (lower quartile (LQ) = 5.9161; upper quartile (UQ) = 6.5745) compared to the Ppre samples (LQ = 5.1224; M = 6.236; UQ = 6.8507) (*p* = 0.6547) (Figure 1A). The Shannon-group difference between the Ppre and Ppost samples indicated no significant differences. The abundance-based coverage-estimator (ACE) index was implemented to calculate the community-species richness in the Ppre and Ppost samples. The outcomes indicate that the community diversity was similar in the Ppre (LQ = 86; M = 150.34; UQ = 167) and Ppost (LQ = 75; M = 97; UQ = 170.35) samples (*p* = 0.6089) (Figure 1B). The dominance was implemented to identify the taxa presented equally in the Ppre and Ppost samples. In that, the greater-dominance indices (*p* > 0.05) indicate that a higher dominance of species may present in the Ppre (LQ = 0.0108; M = 0.024; UQ = 0.0495) than the Ppost (LQ = 0.0149; M = 0.015; UQ = 0.0230) samples (*p* = 0.6547) based on the M-values (Figure 1C). The Simpson evenness was used to measure the diversity accounting for the number of organisms and species in the Ppre and Ppost samples. The results indicate that there was significant diversity in species between the Ppre (LQ = 0.02621; M = 0.311; UQ = 0.5730) and Ppost (LQ = 0.5114; M = 0.5744; UQ = 0.0703) samples (*p* = 0.0254) (Figure 1D).

Likewise, the Shannon index, ACE, dominance, and Simpson evenness were estimated for the Tpre and Tpost samples. In that, the Tpost (LQ = 4.4026; M = 5.023; UQ = 5.4530) M-value was slightly more increased than that of the Tpre (LQ = 3.7693; M = 4.936; UQ = 5.8008) samples. No significant differences were noted between the Tpre and Tpost samples (*p* = 0.8480) (Figure 1E). However, the ACE index showed significant differences between the Tpre and Tpost samples (*p* = 0.0254). The outcomes demonstrate that the community-species richness was significantly more reduced in the Tpost (LQ = 63; M = 120.075; UQ = 156.1651) than the Tpre (LQ = 162.1688; M = 196.202; UQ = 207) samples based on the M-value (Figure 1F). The dominance indicates that there might have been a significant dominant species in Tpre (LQ = 0.0433; M = 0.084; UQ = 0.1987) compared to Tpost (LQ = 0.0426; M = 0.068; UQ = 0.0802) (*p* = 0.5653) (Figure 1G). The estimated Simpson evenness indicates that there were changes in species diversity between the Tpre (LQ = 0.05412; M = 0.064; UQ = 0.1255) and Tpost (LQ = 0.1082; M = 0.1178; UQ = 0.1978) samples (*p* = 0.0639), but the changes were not significant (Figure 1H).

#### 2.2.2. Microbiome Similarity and Variance (Beta-Diversity)

The beta-diversity was assessed using the permutational multivariate analysis of variance (PERMANOVA) with 999 permutations and the *p*-value was estimated to identify the differences between the Ppre vs. Ppost and Tpre vs. Tpost samples. The results of the PERMANOVA indicate that there were no significant differences between the pre-and post-samples of the placebo (pseudo-F = 0.5509; *p* = 0.991) (Figure S1A) and treatment groups (pseudo-F = 0.7777; *p* = 0.699) (Figure S1B) in terms of the beta-diversity of the samples.

Principal coordinate analysis (PCoA) was implemented to determine the microbiome’s similarities and differences using the unweighted UniFrac distances across the placebo and treatment groups in scattered 3D space.

**Figure 2:**
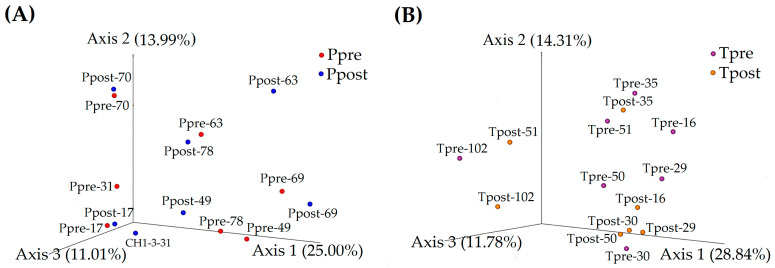
Principal-coordinate-analysis (PCoA) plots for beta-diversity metrics were elucidated to compare the similarity and dissimilarity for the placebo (**A**) and treatment (**B**) samples using PCoA (unweighted UniFrac distances).

In the placebo, PCoA axis 1, axis 2, and axis 3 showed microbial variations of 25.00%, 13.99%, and 11.01%, respectively. In that, samples Ppre-70, Ppost-70, Ppre-17, Ppost-17, Ppre-69, and Ppost-69 were disseminated adjacent to each other, suggesting the existence of common microbial abundances in the respective Ppre and Ppost samples. However, Ppre-31, Ppost-31, Ppre-63, Ppost-63, Ppre-78, Ppost-78, Ppre-49, and Ppost-49 were randomly scattered, demonstrating that these samples might have had some microbial divergence (Figure 2A).

The PCoA analysis for the treatment groups also showed substantial microbial abundances and diversity variations between the Tpre and Tpost samples. Axis 1 (28.84%), axis 2 (14.31%), and axis 3 (11.78%) in the PCoA plot indicated microbial variations in the treatment samples. The Tpre and Tpost samples were scattered separately, except for Tpre-35 and Tpost-35, which were scattered near to each other. The results indicate that the Tpre and Tpost samples could have had certain microbial differences (Figure 2B).

### 2.3. Taxonomy Assignment

#### 2.3.1. Estimation of the Phylum

**Figure 3:**
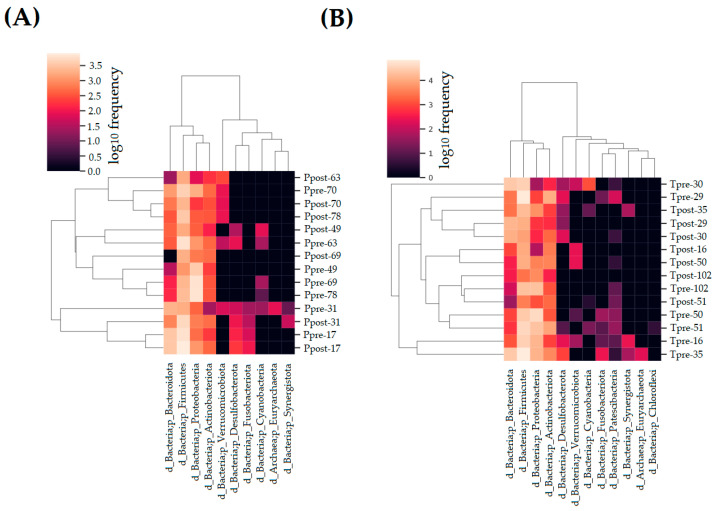
Heat map for the taxonomical assignment of the placebo and treatment samples. A comparison of the estimated phyla in the placebo (**A**) treatment (**B**) samples.

The detected phylum in the placebo and treatment groups is displayed in Figure 3A,B. Ten phyla were found in the placebo group, including *Firmicutes*, *Proteobacteria*, *Bacteroidota*, *Actinobacteriota*, *Verrucomicrobiota*, *Desulfobacterota*, *Fusobaceriota*, *Cyanobacteria*, *Euryarchaeota*, and *Synergistota* (Figure 3A). Twelve phyla, including *Firmicutes*, *Proteobacteria*, *Bacteroidota*, *Actinobacteriota*, *Desulfobacterota*, *Cyanobacteria*, *Verrucomicrobiota*, *Synergistota*, *Fusobaceriota*, *Patescibacteria*, *Euryarchaeota*, and *Chloroflexi*, were detected in the treatment group (Figure 3B).

**Figure 4:**
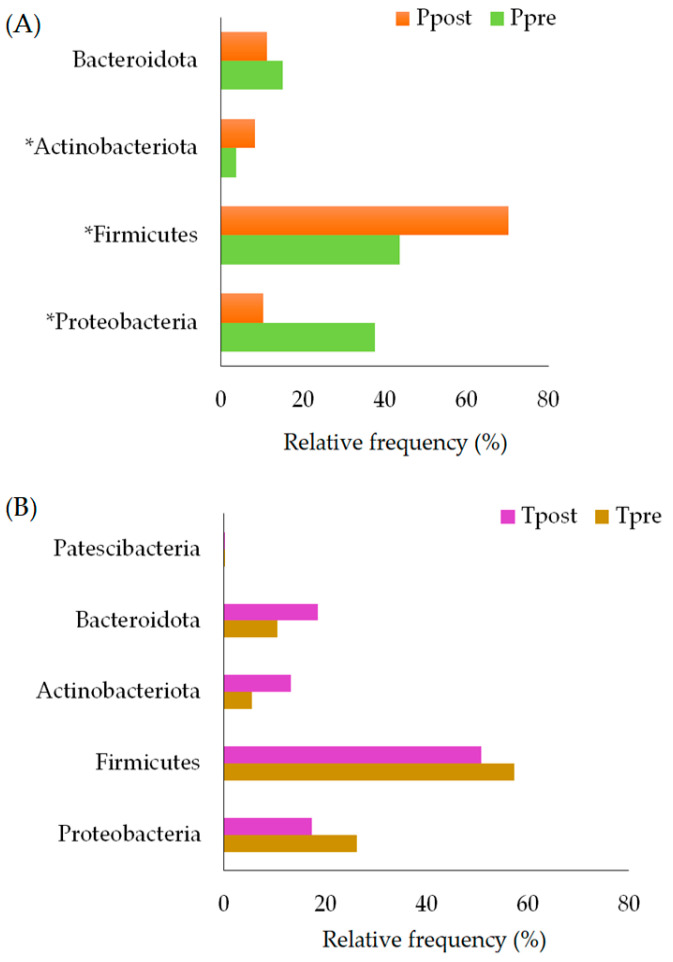
The relative frequency of estimated phyla in the placebo (**A**) treatment (**B**) samples. Ppre: placebo at baseline; Ppost: placebo at week 12; Tpre: treatment at baseline; Tpost: treatment at week 12. * indicates the significant changes.

The relative frequency (RF) of the phylum detected in the placebo (Ppre and Ppost) and treatment (Tpre and Tpost) samples was calculated. The significant inter- and intra-group differences in phyla were estimated in the placebo and treatment samples using the non-parametric Wilcoxon signed-rank and Mann–Whitney test. The results indicate that phyla such as Proteobacteria (*p* = 0.043), Firmicutes (*p* = 0.018), and Actinobacteria (*p* = 0.018) exhibited significant differences in the Ppre and Ppost samples (Figure 4A; Table S1). There were no significant changes between the Tpre and Tpost samples in terms of the abundances of phyla (Figure 4B; Table S2). However, the abundance of phylum Firmicutes was significantly reduced in the Tpost samples compared to the Ppost samples (*p* = 0.025) (Table S3).

#### 2.3.2. Estimation of the Genera

The changes in the microbial composition in placebo and treatment samples are represented in Figure S2A,B.

Non-parametric statistical analysis was performed to predict the significant changes in the detected genera in the placebo and treatment samples. The Wilcoxon signed-ranks test was implemented with a statistical significance of *p* ≤ 0.05. Significant increases in the abundances of *Collinsella* (*p* = 0.022) and *Streptococcus* (*p* = 0.043) were observed in the Ppost samples compared to the Ppre samples (Table S1). Significant decreases in the abundances of *Streptococcus* (*p* = 0.043) and *UBA1819* (*p* = 0.031) were observed in the Tpost samples compared to the Tpre samples (Table S2). The changes in detected genera abundances were insignificant (Tables S1 and S2).

The statistical differences in the genera between the placebo and treatment groups after 12 weeks were calculated using the Mann–Whitney test. The abundances of *Blautia* (*p* = 0.004), *Eubacterium HG* (*p* = 0.013), *Streptococcus* (*p* = 0.025), and *Butyricicoccus* (*p* = 0.018) were significantly reduced in the treatment group compared to the placebo group after 12 weeks of study (Table S3).

#### 2.3.3. Estimation of the Species

The species-level changes in the microbiome of the placebo and treatment samples are presented in Figure S3A,B. The statistical analysis revealed that the abundance of *Shigella* sp. was significantly (*p* = 0.043) reduced in Ppost samples (Table S1). However, no significant changes were detected in the treatment samples between the baseline and 12-week values (Table S2). The abundance of *Eubacterium hallii* (*p* = 0.013) was significantly reduced after 12 weeks in the treatment group compared to the placebo group (Table S3).

## 3. Discussion

Several studies explained the role of gut microbiota in regulating glucose tolerance and insulin sensitivity and improving the symptoms of T2DM. In addition, they reverse the glucose tolerance and fasting glucose in prediabetes [25]. It is well known that gut microbiota play a prominent role in framing the network of host immune functions. In the case of gastrointestinal infections, dietary changes or antibiotics could cause substantial microbiome shifts in individuals. Gut dysbiosis could cause inflammatory, metabolic, and autoimmune disorders [26,27]. Several studies have focused on gut-microbial differences in diabetic and healthy controls, pathophysiology and immunological dysregulation, gut permeability, and the involvement of pathogens in the onset of diabetes [11,28]. The abundance of *Clostridium* was higher in T2DM patients [29]. Modifications of the gut microbiome using probiotics have rendered promising results through various metabolic functions such as improving disease symptoms, limiting damage, and promoting the repair of the gut-epithelial barrier [30].

The supplementation of probiotics (*B. breve* UBBR 01, *Bacillus coagulans* Unique-IS2, *L. salivarius* UBLS22, *L. casei* UBLC42, *L. plantarum* UBLP40, and *L. acidophilus* UBLA34) and a prebiotic (fructo-oligosaccharide) mixture for 12 weeks showed beneficial effects on the FBS, postprandial blood sugar, serum insulin, and homeostatic-model-assessment–insulin-resistance (HOMA-IR) scores. It improved the health-related quality of life of T2DM subjects [31]. The supplementation of a probiotic mixture (*L. acidophilus*, *B. lactis*, *B. bifidum*, and *B. longum*) or synbiotic preparation (probiotics with inulin) for 24 weeks to prediabetic adults reduced fasting plasma glucose, HbA1c, insulin levels, and HOMA-IR score. It increased the quantitative insulin-sensitivity-check index (QUICKI). The study indicated that probiotic or synbiotic supplementation could reduce pre-diabetic people’s risk of developing metabolic disorders [32].

Fasting plasma glucose (FPG), postprandial plasma glucose (PPG), and HbA1c measurement remain important for assessing glycemic control in T2DM patients [33]. HbA1c concentration predicts the complications of diabetes [34,35]. Probiotic supplementation (1 × 10^10^ CFUs of *L. salivarius* subsp. *salicinius* AP-32, *L. johnsonii* MH-68, and *B. animalis* subsp. *lactis* CP-9) improved HbA1c levels in T1DM patients [36]. A multispecies probiotics mix (2 × 10^9^ CFU *L. acidophilus*, 1.5 × 10^9^ CFU *L. rhamnosus*, 7 × 10^9^ CFU *L. casei*, 2 × 10^8^ CFU *L. bulgaricus*, 2 × 10^10^ CFU *B. breve*, 7 × 10^9^ CFU *B. longum*, and 1.5 × 10^9^ CFU *S. thermophilus*) and 100 mg fructo-oligosaccharide supplementation for 8 weeks prevented an increase in FPG, serum hs-CRP, and plasma total glutathione in T2DM patients [37]. The consumption of probiotics (*L. acidophilus* La5 and *B. lactis* Bb12)-based yogurt regulated FPG, HbA1c, and antioxidants in T2DM subjects [38]. Synbiotic supplementation (*L. acidophilus*, *L. plantarum*, *L. fermentum*, *L. gasseri*, and fructooligosaccharides) significantly reduced logTG/HDL-C in T2DM subjects after six weeks [39]. Jiang et al. reported that supplementation with a probiotic mixture (1.2 × 10^9^ CFU *B. bifidum*, 4.2 × 10^9^ CFU *L. acidophilus*, and 4.3 × 10^9^ CFU *S. thermophilus*) for 12 weeks significantly reduced fasting blood glucose (FBG), HbA1c, and microalbuminuria/creatinine (mAlb/Cr) levels in T2DM subjects [40]. Recently, Zhang et al. stated that supplementation with a probiotic preparation (12 weeks; *B. longum*, *L. bulagricumi*, S. *thermophilus*) and insulin effectively reduced the FBG, postprandial-glucose, and LDL-C level in type 1 diabetes-mellitus (T1DM) subjects compared to the placebo [41].

Supplementation with *B. breve* BR03 and B632 improved insulin sensitivity and supported weight loss [42]. Supplementation with multi-strain probiotic preparation containing *B. breve* UBBr01 and other Lactobacillus strains significantly reduced the level of HbA1c and improved the quality of T2DM patients’ life [43]. The current study reports that the body weight, fat, and HbA1c in the *B. breve*-supplemented group were reduced compared to the placebo by the end of the study (Table 3). However, intra-group analysis showed no significant changes in the clinical parameters in the treatment group (Table 2).

Significant changes were observed in body-fat, ALP, HbA1c, and HDL values in the placebo group, whereas creatinine, ALP, HDL, and LDL values were changed in the treatment group. However, comparing the differences in changes in both the placebo and treatment groups after 12 weeks of study, body weight and fat, FBS, and HbA1c showed significant changes. Additionally, HbA1c and body fat were negatively improved in the placebo group, and the HDL and ALP changes were insignificant compared to the treatment group. Accordingly, *B. breve* supplementation could prevent the worsening of the clinical conditions. Further studies (with more participants, prolonged intervention periods, and follow-ups) are needed to explain the changes in the placebo and treatment groups (Table 2).

Next-generation sequencing studies revealed that *L. salivarius* subsp. *Salicinius* AP-32, *L. johnsonii* MH-68, and *B. animalis* subsp. *lactis* CP-9 supplementation enriched the gut microbiota, especially *B. animalis*, *L. salivarius*, and *Akkermansia muciniphila*, and reduced FBG and HbA1c in T1DM patients [38]. *L. reuteri* ADR-1 and ADR-3 supplementation increased the abundance of *Bifidobacterium* spp., and *Lactobacillus* spp. *L. reuteri* ADR-3 consumption was positively correlated with the abundance of Firmicutes [44].

Firmicutes and Bacteroidetes dominate about 80–90% of gut microbiota. Firmicutes are the main phyla in healthy individuals, whereas Bacteroidetes may disturb gut-barrier function, induce inflammation, and promote gut leakiness by inhibiting the production of tight-junction proteins [45]. Other major phyla in a healthy human gut are Actinobacteria, Verrucomicrobia, and Proteobacteria [46,47]. The Firmicutes-to-Bacteroidetes (F/B) ratio could be suggested as an index of healthy gut microbiota [48]. In both T2DM and healthy subjects, Bacteroidetes was the dominant phylum. Among the phylum Bacteroidetes, the abundance of the genus *Bacteroides* was higher, whereas the abundance of the genus *Prevotella* was lower in T2DM subjects, which was vice versa in healthy subjects [49]. The ratio of Firmicutes/Bacteroidetes was higher in T2D patients than in healthy subjects [50]. The study of gut microbiota in T2DM showed that the class Clostridia and phylum Firmicutes declined, and members of the class Betaproteobacteria were enhanced and associated with plasma glucose. The Firmicutes-to-Bacteroidetes ratio is positively related to plasma glucose in T2DM [1]. The previous findings do not support the changes in the abundance of Firmicutes and *Bacteroides* observed in the present study (Tables S2 and S3). The insignificant changes and enigmatic results may be due to the fewer study subjects. However, the Firmicutes and Bacteroidetes ratio changes were associated with changes in body weight and BMI [51,52].

T2DM subjects were reported to have alterations in the abundances of *Bacteroides*, Firmicutes, *Prevotella*, and Actinobacteriota [49,53]. Our results show that Actinobacteriota, Firmicutes, and Proteobacteria were significantly altered in the placebo group after 12 weeks (Figure 4A, Table S1). However, the above-mentioned phyla were not affected significantly after *B. breve* intervention in T2DM subjects (Figure 4B, Table S2).

It is unlikely that changes observed in the microbiome of the treatment group (Tables S1–S3; Figure S2) are correlated with improvements in body weight, fat, FBS, or HbA1c because there was no significant augmentation in the clinical parameters and the sample size evaluated was too small to conclude.

The genus *Blautia* was increased in T2DM and obese subjects with non-alcoholic fatty liver [54,55,56]. Moreover, *Streptococcus* abundance was positively associated with T2DM and higher BMI [57,58]. *Butyricicoccus* was associated with weight loss in the energy-restricted Mediterranean-diet subjects [59]. The abundance of *Blautia* (*p* = 0.004), *Streptococcus* (*p* = 0.025), *Butyricicoccus* (*p* = 0.018), and *Eubacterium HG* (*p* = 0.013) was significantly reduced in the treatment group (Table S3), indicating that *B. breve* supplementation could improve the gut microbiota.

Metagenomic analysis showed that T2DM subjects in China have a higher abundance of *Escherichia coli*, *Bacteroides caccae*, and *Clostridium* species and *Eggerthella lenta*, and less SCFA-producing *Roseburia intestinalis*, *R. inulinivorans*, *Eubacterium rectale*, *Faecalibacterium prausnitzii*, and *Clostridiales* sp. *SS3/4*. High abundance in *Lactobacillus gasseri* and *Streptococcus mutans*, few *Clostridiales* sp., and reduced SCFA-producing *Roseburia*, *Eubacterium eligens*, and *Bacteroides intestinalis* was demonstrated in T2DM subjects in Europe [60]. No significant changes were found at the species level in the Tpost sample compared to the Tpre samples. However, a significant change in *Shigella* sp. was observed in Ppost samples (Figure S3). A significant change was observed in the abundance of *Eubacterium hallii* (*p* = 0.013) in the treatment group (Table S3). The rationale for the changes in *E. hallii* and *Shigella* sp. remains to be elucidated.

Several studies reported the effect of probiotic supplementation on the health profile of T2DM subjects, but the results could be more consistent. Such inconsistency might be associated with single or multiple strains, doses, study duration, and ethnicity. The current study has some limitations, such as fewer subjects, the use of a single probiotic strain, no dietary restrictions, no record of physical activity, the duration of the study, and fewer metagenomic samples for microbiome analysis. Therefore, the results of the current study require further validation using more experimental subjects.

## 4. Materials and Methods

The study protocols were approved by the Ethical Committee regulations of Phrae Provincial Public Health Office, Phrae, Thailand (approval number: PPH No.1/2562), and the study was carried out accordingly. Before commencement of the study, the purpose and methodology of the study were described to the subjects, and they provided written consent before the study.

### 4.1. Study Subjects

A randomized, double-blind, placebo-controlled clinical trial was performed. Adult T2DM subjects aged 20–70 years were included in the study. Subjects with abnormal liver or renal function; malignant, micro, or macrovascular complications; heavy alcoholism and smoking; pregnancy; feeding stage; medication (non-steroidal, anti-inflammatory); and other discomforts were excluded from the study. Subjects were excluded if they had had antibiotic treatment within 14 days leading up to the study. The researchers and participants were completely blinded to the supplements. Participants were randomized to receive either placebo or a probiotic supplement for 12 weeks. Blood and fecal samples of the participants were analyzed at baseline (week 0) and after 12 weeks of supplementation. Participants were asked to perform assigned follow-up visits without absence. The intra and inter-group microbial-diversity variations were analyzed from fecal samples. The samples collected at baseline were designated as Ppre (placebo at baseline) and Tpre (treatment at baseline); likewise, samples collected after 12 weeks were denoted as Ppost (placebo at week 12) and Tpost (treatment at week 12). A power analysis was performed to estimate the minimum sample size needed for the study, with a power of 0.80 and a drop-out rate of ~32%.

### 4.2. Study Protocol

**Figure 5:**
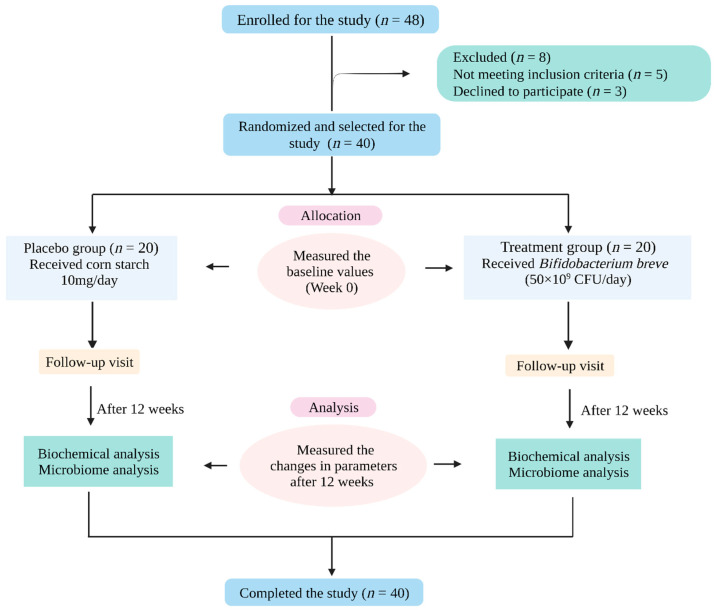
Schematic representation of the study protocol (figure created using BioRender.com (accessed on 4 January 2023)).

The participants (*n* = 40) were randomly assigned to placebo and probiotic groups at a ratio of 1:1. Aluminum-foil sachets containing corn starch and probiotic supplement were given to participants of the respective groups. Participants in the placebo group received corn starch (10 mg/day), and the probiotic group received probiotic *Bifidobacterium breve* (LACTOMASON company, Jinju, Korea) (50 × 10^9^ CFU/day) for 12 weeks. The study plan is schematically represented in Figure 5. They were asked to store the sachets in the refrigerator (4–6 °C). They were instructed to consume the content of one sachet per day by mixing it in drinking water 20 min before dinner. During the study, participants were informed to strictly avoid fermented foods or other dietary supplements.

### 4.3. Demographic Assessments

The sociodemographic and clinical data were collected from the participants through personal consultation. The participants’ history, including physical activity and habits such as smoking, drinking, and medication, was assessed. Afterwards, the participants were randomly assigned into two groups, placebo (*n* = 20) or probiotic (*n* = 20). Their basic characteristics, such as age, gender, smoking, and alcohol intake, were recorded. Other characteristics, such as body weight, BMI, body fat, and visceral fat, were measured using a weighing scale (Picooc^®^, Model S1 Pro, Beijing, China).

### 4.4. Blood Biochemical Analysis

Blood samples were collected from the participants after overnight fasting at week 0 and week 12 of the study in ethylenediaminetetraacetic acid (EDTA)-coated containers and stored at 4 °C until use. The samples were centrifuged at 1000× *g* for 15 min at 2–8 °C. Plasma samples were carefully separated into pyrogen-free tubes and stored at −80 °C for further analysis. Blood biochemical parameters such as fasting blood sugar (FBS), blood-urea nitrogen (BUN), creatinine, aspartate aminotransferase (AST), alanine aminotransferase (ALT), alkaline phosphatase (ALP), HbA1c, total cholesterol (TC), triglyceride (TG), low-density cholesterol (LDL), and high-density cholesterol (HDL) were evaluated at the Clinical Laboratory Center (CLC, Mueang, Phrae district, Phrae-54000).

### 4.5. Next-Generation Sequencing

Stool samples were collected from the participants at week 0 and week 12 using a sterile container. According to the manufacturer protocol, bacterial genomic DNA was extracted from the fecal samples using a QIAmp UCP DNA Micro Kit (Catalog no. 56204, QIAGEN, Hilden, Germany). The Omics Sciences and Bioinformatics Center, Faculty of Science, Chulalongkorn University, Thailand, performed the sequencing analysis. The variable regions of the 16s RNA gene (V3–V4) were amplified and sequenced using the Illumina MiSeq platform next-generation sequencing system (Illumina, Inc., 5200 Illumina Way, San Diego, CA, USA) as detailed previously [61].

The valid sequences were identified by matching the raw sequences with the barcode of the corresponding sequences. The raw-sequence tags were analyzed using Quantitative Insights Into Microbial Ecology (QIIME 2^TM^). After chimera detection, the sequences were clustered into operational taxonomic units (OTUs) with 97% sequence identity. After checking the quality of pair-end reads with the information in FASTQ files using DADA2, the poor-quality reads were filtered out as per the default QIIME 2™ threshold values (minimum quality score = 25, minimum/maximum length = 200/1000, no ambiguous bases allowed, and no mismatches allowed in the primer sequence).

The paired-end data were acquired for each sample as forward and reverse read in two different FASTQ files, which were then paired using the 16S rRNA methodology of QIIME2.0^TM^. The Shannon-diversity index was ascertained to estimate the significant differences between the groups using the Kruskal–Wallis (pairwise) test for the placebo (*n* = 7) and treatment (*n* = 7) samples. Principal coordinate analysis (PCoA) was used to determine the relationship between the samples, and QIIME 2™ View was used to display it. Here, PCoA plots were made using the first three principal coordinates, labeled according to their variance. The phylum, genus, and species diversity of the placebo- and treatment-group samples were compared separately (Ppre vs. Ppost; Tpre vs. Tpost and Ppro vs. Tpro; Ppost vs. Tpost). PERMANOVA analysis was performed using QIIME 2^TM^ as per the previous report [62].

### 4.6. Statistical Analysis

The demographic parameters were analyzed using the independent *t*-, Fisher, and Mann–Whitney tests. Power analysis was performed using STATA Statistical software version 15.1 (Brazos County, TX, USA).

Data were analyzed using STATA Statistical software version 15.1 (Brazos County, TX, USA). Data are represented as mean ± SD. The biochemical variables and microbiome differences were analyzed using the paired *t*-test and the Wilcoxon signed-rank test. The changes in placebo and treatment groups were compared using the Mann–Whitney test.

Gaussian-regression analysis was conducted for the treatment group to differentiate the parameters between baseline and after 12 weeks of study. The minimum level of statistical significance was set as *p* < 0.05.

The significant changes in fecal microbial diversity between the groups regarding phylum, genus, and species were estimated using the Wilcoxon signed-rank test (intra-group) and Mann–Whitney test (inter-group). Power analysis was performed to confirm the validity of the statistical analysis.

## 5. Conclusions

Supplementation with *B. breve* for 12 weeks prevented a worsening of the studied clinical parameters in T2DM subjects. The changes observed in the placebo group were unexplainable because of the small number of subjects. The changes in clinical parameters and microbiome in the treatment group were questionable because of the small number of subjects. We strongly endorse that further detailed, in-depth studies are mandatory to confirm the results of the present study, which may help develop probiotic-based supplements to treat diabetic conditions.

## Figures and Tables

**Table 1:** The sociodemographic and clinical characteristics of the study participants.

No	Parameters	Placebo (*n* = 20)	Treatment (*n* = 20)	*p*-Value
1	Age (year)	61.05 ± 1.83	63.90 ± 1.44	0.228 ^a^
2	Gender			1.000 ^b^
	Male	3 (15.00)	3 (15.00)	
	Female	17 (85.00)	17 (85.00)	
3	Smoking			
	No	20 (100.00)	20 (100.00)	
	Yes	0 (00.00)	0 (00.00)	
4	Alcohol consumption			1.000 ^b^
	No	19 (95.00)	19 (95.00)	
	Yes	1 (5.00)	1 (5.00)	
5	Body weight (kg)	58.99 ± 2.37	54.86 ± 2.17	0.207 ^a^
6	BMI (kg/m^2^)	25.73 ± 1.27	23.80 ± 0.74	0.196 ^a^
7	Body fat (%)	28.21 ± 1.59	26.11 ± 1.27	0.308 ^a^
8	Visceral fat (%)	12.05 ± 0.93	10.00 ± 0.67	0.082 ^a^
9	BUN (mg/dL)	18.44 ± 2.07	14.69 ± 0.86	0.102 ^a^
10	Creatinine (mg/dL)	1.45 ± 0.13	1.28 ± 0.06	0.235 ^a^
11	AST (IU/L)	21.89 ± 1.91	24.65 ± 3.12	0.988 ^c^
12	ALT (IU/L)	22.11 ± 2.93	21.60 ± 3.30	0.403 ^c^
13	ALP (IU/L)	102.17 ± 4.82	98.67 ± 5.84	0.647 ^a^
14	FBS (mg/dL)	142.40 ± 9.86	148.25 ± 10.74	0.691 ^a^
15	HbA1c (%)	6.85 ± 0.37	7.44 ± 0.49	0.343 ^a^
16	TC (mg/dL)	192.85 ± 9.69	196.40 ± 8.20	0.781 ^a^
17	TG (mg/dL)	158.80 ± 18.38	156.25 ± 16.51	0.978 ^c^
18	HDL (mg/dL)	57.85 ± 2.55	63.75 ± 4.40	0.253 ^a^
19	LDL (mg/dL)	102.94 ± 7.14	113.61 ± 10.04	0.392 ^a^

BMI: body-mass index; BUN: blood-urea nitrogen; AST: aspartate aminotransferase; ALT: alanine transaminase; ALP: alkaline phosphatase; FBS: fasting blood sugar, HbA1c: glycated hemoglobin; TC: total cholesterol; TG: triglycerides; HDL: high-density lipoprotein; LDL: low-density lipoprotein. Data are mean ± SD. ^a^ *p*-value from the independent *t*-test, ^b^ *p*-value from Fischer’s exact test, ^c^ *p*-value from the Mann–Whitney test.

**Table 2:** Biochemical parameters at baseline (week 0) and end of the study (week 12) in the placebo and treatment groups.

Parameters	Placebo (*n* = 20)		*p*-Value	Treatment (*n* = 20)		*p*-Value
	Ppre	Ppost		Tpre	Tpost	
Body weight (kg)	58.99 ± 2.37	60.35 ± 2.23	0.061 ^a^	54.86 ± 2.17	54.45 ± 2.15	0.466 ^a^
BMI (kg/m^2^)	25.73 ± 1.27	26.24 ± 1.15	0.095 ^a^	23.80 ± 0.74	23.54 ± 0.69	0.269 ^a^
Body fat (%)	28.21 ± 1.59	32.06 ± 1.54	<0.001 *^a^	26.11 ± 1.27	26.09 ± 1.18	0.989 ^a^
Visceral fat (%)	12.05 ± 0.93	12.45 ± 0.87	0.119 ^a^	10.00 ± 0.67	9.40 ± 0.50	0.244 ^a^
BUN (mg/dL)	18.44 ± 2.07	17.65 ± 2.34	0.412 ^b^	14.69 ± 0.86	13.15 ± 0.56	0.083 ^b^
Creatinine (mg/dL)	1.45 ± 0.13	1.25 ± 0.12	0.226 ^a^	1.28 ± 0.06	1.04 ± 0.05	<0.001 *^a^
AST (IU/L)	21.89 ± 1.91	24.61 ± 2.85	0.230 ^b^	24.65 ± 3.12	23.45 ± 2.40	0.561 ^b^
ALT (IU/L)	22.11 ± 2.93	29.61 ± 10.08	0.391 ^b^	21.60 ± 3.30	20.35 ± 2.76	0.464 ^b^
ALP (IU/L)	102.17 ± 4.82	90.61 ± 10.87	0.003 *^b^	98.67 ± 5.84	81.56 ± 3.49	0.001 *^b^
FBS (mg/dL)	142.40 ± 9.86	153.65 ± 9.69	0.114 ^a^	148.25 ± 10.74	141.65 ± 8.84	0.381 ^a^
HbA1c (%)	6.85 ± 0.37	7.61 ± 0.47	0.016 *^a^	7.44 ± 0.49	7.34 ± 0.55	0.732 ^a^
TC (mg/dL)	192.85 ± 9.69	195.50 ± 9.21	0.770 ^a^	196.40 ± 8.20	192.10 ± 9.97	0.467 ^a^
TG (mg/dL)	158.80 ± 18.38	183.30 ± 19.56	0.065 ^b^	156.25 ± 16.51	157.95 ± 13.82	0.896 ^b^
HDL (mg/dL)	57.85 ± 2.55	65.85 ± 4.14	0.026 *^a^	63.75 ± 4.40	77.00 ± 5.25	0.027 *^a^
LDL (mg/dL)	102.94 ± 7.14	104.97 ± 9.61	0.601 ^b^	113.61 ± 10.04	95.64 ± 10.61	0.009 *^b^

Ppre: placebo at baseline; Ppost: placebo at week 12; Tpre: treatment at baseline; Tpost: treatment at week 12; BMI: body-mass index; BUN: blood-urea nitrogen; AST: aspartate aminotransferase; ALT: alanine transaminase; ALP: alkaline phosphatase; FBS: fasting blood sugar, HbA1c: glycated hemoglobin; TC: total cholesterol; TG: triglycerides; HDL: high-density lipoprotein; LDL: low-density lipoprotein. Data are mean ± SD. * indicates the significant difference in *p*-value at a 95% confidence interval. ^a^ *p*-value from paired *t*-test, ^b^ *p*-value from the Wilcoxon signed-rank test.

**Table 3:** The differentiation in biochemical parameters between the placebo and treatment groups at the end of the study.

Variables	Difference		*p*-Value
	Placebo (*n* = 20)	Treatment (*n* = 20)	
Body weight (kg)	1.36 ± 0.68	−0.41 ± 0.55	0.023 *
BMI (kg/m^2^)	0.51 ± 0.29	−0.26 ± 0.23	0.060
Body fat (%)	3.86 ± 0.59	−0.01 ± 1.05	0.004 *
Visceral fat (%)	0.40 ± 0.24	−0.60 ± 0.50	0.080
BUN (mg/dL)	−0.80 ± 1.56	−1.55 ± 0.81	0.735
Creatinine (mg/dL)	−0.20 ± 0.16	−0.25 ± 0.04	0.715
AST (IU/L)	2.72 ± 1.94	−1.20 ± 2.75	0.628
ALT (IU/L)	7.50 ± 10.25	−1.25 ± 2.70	0.326
ALP (IU/L)	−11.56 ± 11.34	−17.11 ± 3.37	0.635
FBS (mg/dL)	11.25 ± 6.79	−6.60 ± 7.36	0.044 *
HbA1c (%)	0.76 ± 0.28	−0.10 ± 0.29	0.023 *
TC (mg/dL)	2.65 ± 8.94	−4.30 ± 5.79	0.351
TG (mg/dL)	24.50 ± 12.82	1.70 ± 21.00	0.449
HDL (mg/dL)	8.00 ± 3.32	13.25 ± 5.53	0.808
LDL (mg/dL)	2.03 ± 11.69	−17.97 ± 5.88	0.337

BMI: body-mass index; BUN: blood-urea nitrogen; AST: aspartate aminotransferase; ALT: alanine transaminase; ALP: alkaline phosphatase; FBS: fasting blood sugar, HbA1c: glycated hemoglobin; TC: total cholesterol; TG: triglycerides; HDL: high-density lipoprotein; LDL: low-density lipoprotein. Data are mean ± SE. * indicates the significant difference in *p*-value at a 95% confidence interval.

**Table 4:** Gaussian-regression analysis of the treatment group after 12 weeks of study.

Parameters	Coefficient	95% Confidence Interval	*p*-Value
Body weight (kg)	−2.52	−6.50 to 1.46	0.198
BMI (kg/m^2^)	−1.08	−2.71 to 0.55	0.178
Body fat (%)	−3.32	−7.19 to 0.55	0.088
Visceral fat (%)	−1.62	−2.92 to 0.31	0.018 *
BUN (mg/dL)	−3.08	−7.60 to 1.45	0.171
Creatinine (mg/dL)	−0.12	−0.33 to 0.08	0.218
AST (IU/L)	−1.82	−8.01 to 4.37	0.553
ALT (IU/L)	−6.48	−27.69 to 14.73	0.538
ALP (IU/L)	−4.88	−29.86 to 20.09	0.692
FBS (mg/dL)	−22.95	−42.25 to −3.66	0.021 *
HbA1c (%)	−1.35	−2.43 to −0.28	0.016 *
TC (mg/dL)	−28.89	−56.61 to −1.17	0.042 *
TG (mg/dL)	−46.44	−101.33 to 8.46	0.094
HDL (mg/dL)	8.61	−4.71 to 21.92	0.198
LDL (mg/dL)	−28.84	−56.48 to −1.20	0.041 *

BMI: body-mass index; BUN: blood-urea nitrogen; AST: aspartate aminotransferase; ALT: alanine transaminase; ALP: alkaline phosphatase; FBS: fasting blood sugar, HbA1c: glycated hemoglobin; TC: total cholesterol; TG: triglycerides; HDL: high-density lipoprotein; LDL: low-density lipoprotein. Data are mean ± SD. * indicates the significant difference in *p*-value at a 95% confidence interval.

## Data Availability

The data presented in this study are available within the article.

## Supplementary Materials

The following supporting information can be downloaded at: https://www.mdpi.com/article/10.3390/ph16050695/s1, Figure S1: The PERMANOVA was estimated to identify the distances between the Ppre vs. Ppost, and Tpre vs. Tpost samples in the placebo and treatment groups (Statistical significance *p* < 0.001); Figure S2: The relative frequency of estimated genera in the placebo (A) treatment (B) samples. Ppre: Placebo at baseline; Ppost: Placebo at week 12; Tpre: Treatment at baseline; Tpost: Treatment at week 12; Figure S3: The relative frequency of estimated species in the placebo (A) treatment (B) samples. Ppre: Placebo at baseline; Ppost: Placebo at week 12; Tpre: Treatment at baseline; Tpost: Treatment at week 12; Table S1: The statistical differences in the phylum, genus, and species between the Ppre and Ppost samples; Table S2: The statistical differences in the phylum, genus, and species between the Tpre and Tpost samples; Table S3: The statistical differences in the phylum, genus, and species level between the placebo and treatment groups after 12 weeks of study.
