# Supplementary material for: Influence of Bifidobacterium breve on the Glycaemic Control, Lipid Profile and Microbiome of Type 2 Diabetic Subjects: A Preliminary Randomized Clinical Trial
Source: Pharmaceuticals (Basel). 2023 May 4;16(5):695. doi: 10.3390/ph16050695 (PMC10220806; doi:10.3390/ph16050695)
Supplement: Supplementary file 1 [file pharmaceuticals-16-00695-s001.zip › pharmaceuticals-2232379-supplementary.pdf]

Article

Influence of *Bifidobacterium breve* on the Glycaemic Control, Lipid Profile and Microbiome of Type 2 Diabetic Subjects: A Preliminary Randomized Clinical Trial

Chaiyavat Chaiyasut [1,*], Bhagavathi Sundaram Sivamaruthi [1,2], Narissara Lailerd [1,3], Sasithorn Sirilun [1], Subramanian Thangaleela [1], Suchanat Khongtan [1], Muruganantham Bharathi [1], Periyanaina Kesika [1,2], Manee Saelee [4], Thiwanya Choeisoongnern [4], Pranom Fukngoen [1], Sartjin Peerajan [5] and Phakkharawat Sittiprapaporn [4]

[1] Innovation Center for Holistic Health, Nutraceuticals, and Cosmeceuticals, Faculty of Pharmacy, Chiang Mai University, Chiang Mai 50200, Thailand; sivamaruthi.b@cmu.ac.th (B.S.S.)
[2] Office of Research Administration, Chiang Mai University, Chiang Mai 50200, Thailand
[3] Department of Physiology, Faculty of Medicine, Chiang Mai University, Chiang Mai 50200, Thailand
[4] Neuropsychological Research Laboratory, Neuroscience Research Center, School of Anti-Aging and Regenerative Medicine, Mae Fah Luang University, Bangkok 10110, Thailand; wichian.sit@mfu.ac.th (P.S.)
[5] Health Innovation Institute, Chiang Mai 50200, Thailand
* Correspondence: chaiyavat@gmail.com; Tel.: +66-53-944-340

Supplementary tables and figures:

Table S1. The statistical differences in the phylum, genus, and species between the Ppre and Ppost samples.

Taxonomy	Ppre	Ppost	*p*-value
Phylum			
Proteobacteria	37.67 ± 11.71	10.27 ± 2.17	0.043*
Firmicutes	43.68 ± 7.75	70.20 ± 3.31	0.018*
Actinobacteriota	3.63 ± 0.72	8.23 ± 1.53	0.018*
Bacteroidota	15.02 ± 7.08	11.30 ± 3.50	0.735
Genus			
Shigella	48.24 ± 13.49	17.18 ± 4.29	0.063
Blautia	13.83 ± 7.65	21.35 ± 4.35	0.237
Collinsella	1.97 ± 0.51	9.13 ± 2.58	0.022*
Eubacterium hallii group	2.78 ± 0.79	6.39 ± 1.95	0.063
Eubacterium coprostanoligenes group	3.35 ± 2.04	8.04 ± 2.93	0.203
Streptococcus	4.75 ± 1.47	11.61 ± 5.20	0.043*
Bacteroides	19.43 ± 9.71	18.25 ± 7.02	0.499
Ruminococcus	2.19 ± 0.63	4.22 ± 1.63	0.445
Butyricicoccus	2.08 ± 0.77	2.67 ± 0.49	0.445
Veillonella	1.39 ± 0.57	1.15 ± 0.47	0.553
Species			
Shigella sp.	56.68 ± 12.29	23.82 ± 5.40	0.043*
Eubacterium hallii	3.80 ± 1.16	8.63 ± 2.46	4.83

Bacteroides vulgatus	7.88 ± 4.35	8.58 ± 6.22	0.70

Data are mean ± SD. Ppre: Placebo at baseline; Ppost: Placebo at week 12. The Wilcoxon signed-rank test was used to determine the statistical significance. * Statistically significant if $p \leq 0.05$.

Table S2. The statistical differences in the phylum, genus, and species between the Tpre and Tpost samples.

**Taxonomy**	**Tpre**	**Tpost**	***P*-value**
Phylum			
Proteobacteria	26.33 ± 8.74	17.35 ± 7.65	0.499
Firmicutes	57.41 ± 5.25	50.83 ± 5.62	0.499
Actinobacteriota	5.58 ± 2.15	13.18 ± 4.28	0.128
Bacteroidota	10.63 ± 6.25	18.60 ± 9.04	0.237
Patescibacteria	0.05 ± 0.02	0.04 ± 0.03	0.311
Genus			
Shigella	28.28 ± 9.68	18.35 ± 8.02	0.499
Faecalibacterium	6.57 ± 3.30	10.26 ± 2.39	0.237
Collinsella	3.46 ± 1.56	5.77 ± 2.85	0.237
Blautia	9.58 ± 2.99	5.32 ± 1.97	0.398
Streptococcus	5.56 ± 2.71	1.87 ± 1.15	0.043*
Clostridium	6.26 ± 3.83	3.26 ± 1.45	0.612
Bacteroides	4.78 ± 2.78	11.96 ± 5.59	0.237
Prevotella	6.50 ± 5.47	7.01 ± 4.97	0.932
Eubacterium	2.43 ± 1.06	3.08 ± 1.69	0.799
Eubacterium CG	2.43 ± 1.06	3.08 ± 1.69	0.799
Eubacterium HG	3.52 ± 1.07	1.20 ± 0.46	0.063
Eubacterium EG	0.15 ± 0.10	0.21 ± 0.14	0.263
Subdoligranulum	2.23 ± 1.03	3.26 ± 1.31	0.672
Enterococcus	2.94 ± 1.92	0.77 ± 0.31	0.445
Dorea	1.88 ± 0.82	1.22 ± 0.22	0.866
Monoglobus	1.39 ± 0.84	0.39 ± 0.33	0.398
Bifidobacterium	0.43 ± 0.24	7.13 ± 3.39	0.176
Weissella	0.86 ± 0.29	3.75 ± 3.05	1.000
Anaerostipes	1.20 ± 0.62	0.71 ± 0.20	0.499
Lactococcus	0.24 ± 0.07	2.78 ± 1.70	0.866
UCG-002	0.87 ± 0.67	0.77 ± 0.48	0.672
Romboutsia	2.34 ± 1.37	2.35 ± 1.10	0.672
Roseburia	0.95 ± 0.51	0.61 ± 0.42	0.612
NK4A136	0.74 ± 0.44	0.59 ± 0.20	0.799
Coprococcus	0.94 ± 0.50	1.32 ± 0.83	0.672
Ruminococcus	0.86 + 0.39	0.51 + 0.16	0.799
Incertae Sedis	0.83 ± 0.33	1.31 ± 0.70	0.499
Butyricicoccus	0.43 ± 0.36	0.16 ± 0.04	0.499
Alistipes	0.69 ± 0.48	0.37 ± 0.15	0.398
Fusicatenibacter	0.46 ± 0.15	1.19 ± 0.58	0.445
Slackia	0.24 ± 0.10	0.36 ± 0.13	0.128
R-7	0.26 ± 0.06	0.34 ± 0.20	0.932
Veillonella	0.55 ± 0.19	0.27 ± 0.18	0.270
Desulfovibrio	0.21 ± 0.07	0.60 ± 0.43	0.866

UBA1819	0.27 ± 0.16	0.03 ± 0.03	0.031*
Family_XIII_AD3011	0.15 ± 0.10	0.21 ± 0.14	0.263
Species			
Shigella sp.	32.36 ± 10.40	22.56 ± 8.53	0.612
Streptococcus salivarius	3.90 ± 3.12	0.82 ± 0.51	0.612
Clostridium sensu stricto sp.	5.17 ± 3.39	0.46 ± 0.22	0.091
Eubacterium hallii	4.36 ± 1.17	1.76 ± 0.68	0.091
Lactococcus garvieae	0.21 ± 0.05	3.58 ± 2.22	0.499
Bacteroides vulgatus	0.98 ± 0.61	1.63 ± 0.74	0.051
NK4A136 sp.	1.29 ± 0.71	1.94 ± 1.22	0.672
Ruminococcus torques	0.52 ± 0.14	1.48 ± 0.66	0.499
Bacteroides ovatus	0.05 ± 0.01	3.27 ± 2.70	0.176

Data are mean ± SD. Tpre: Treatment at baseline; Tpost: Treatment at week 12. The Wilcoxon signed-rank test was used to determine the statistical significance. * Statistically significant if $p \leq 0.05$.

Table S3. The statistical differences in the phylum, genus, and species level between the placebo and treatment groups after 12 weeks of study.

**Taxonomy**	**Placebo Vs. Treatment**		***p*-value**
Phylum			
Proteobacteria	10.27 ± 2.17	17.35 ± 7.65	0.848
Firmicutes	70.20 ± 3.31	50.83 ± 5.62	0.025*
Actinobacteriota	8.23 ± 1.53	13.18 ± 4.28	0.749
Bacteroidota	11.30 ± 3.50	18.60 ± 9.04	0.949
Genus			
Shigella	17.18 ± 4.29	18.35 ± 8.02	0.848
Blautia	21.35 ± 4.35	5.32 ± 1.97	0.004*
Collinsella	9.13 ± 2.58	5.77 ± 2.85	0.338
Eubacterium HG	6.39 ± 1.95	1.20 ± 0.46	0.013*
Eubacterium CG	8.04 + 2.93	3.08 + 1.69	0.178
Streptococcus	11.61 ± 5.20	1.87 ± 1.15	0.025*
Bacteroides	18.25 ± 7.02	11.96 ± 5.59	0.655
Ruminococcus	4.22 ± 1.63	1.31 ± 0.70	0.223
Butyricicoccus	2.67 ± 0.49	0.37 ± 0.15	0.018*
Veillonella	1.15 ± 0.47	0.60 ± 0.43	0.375
Species			
Shigella sp.	23.82 ± 5.40	22.56 ± 8.53	0.655
Eubacterium hallii	8.63 ± 2.46	1.76 ± 0.68	0.013*
Bacteroides vulgatus	8.58 ± 6.22	1.63 ± 0.74	0.796

Data are mean ± SD. The Mann-Whitney test was used to determine the statistical significance. * Statistically significant if $p \leq 0.05$.

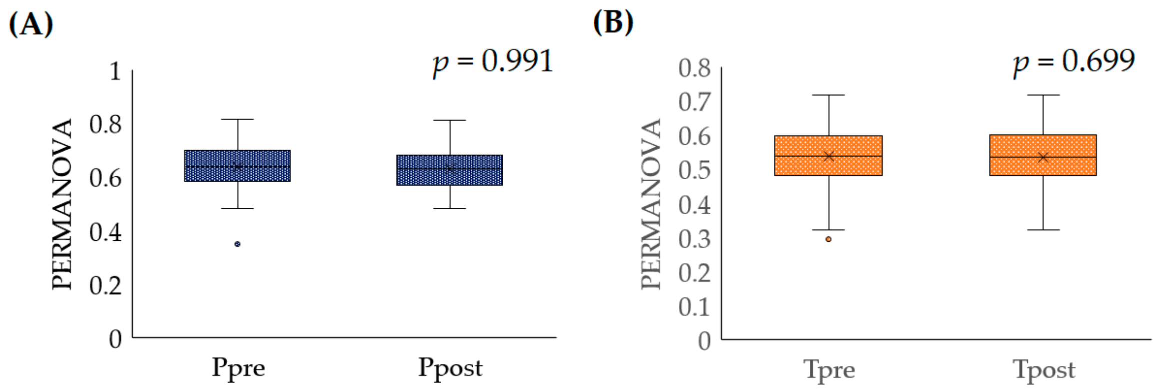

Figure S1: The PERMANOVA was estimated to identify the distances between the Ppre Vs. Ppost, and Tpre Vs. Tpost samples in the placebo and treatment groups (Statistical significance $p < 0.001$).

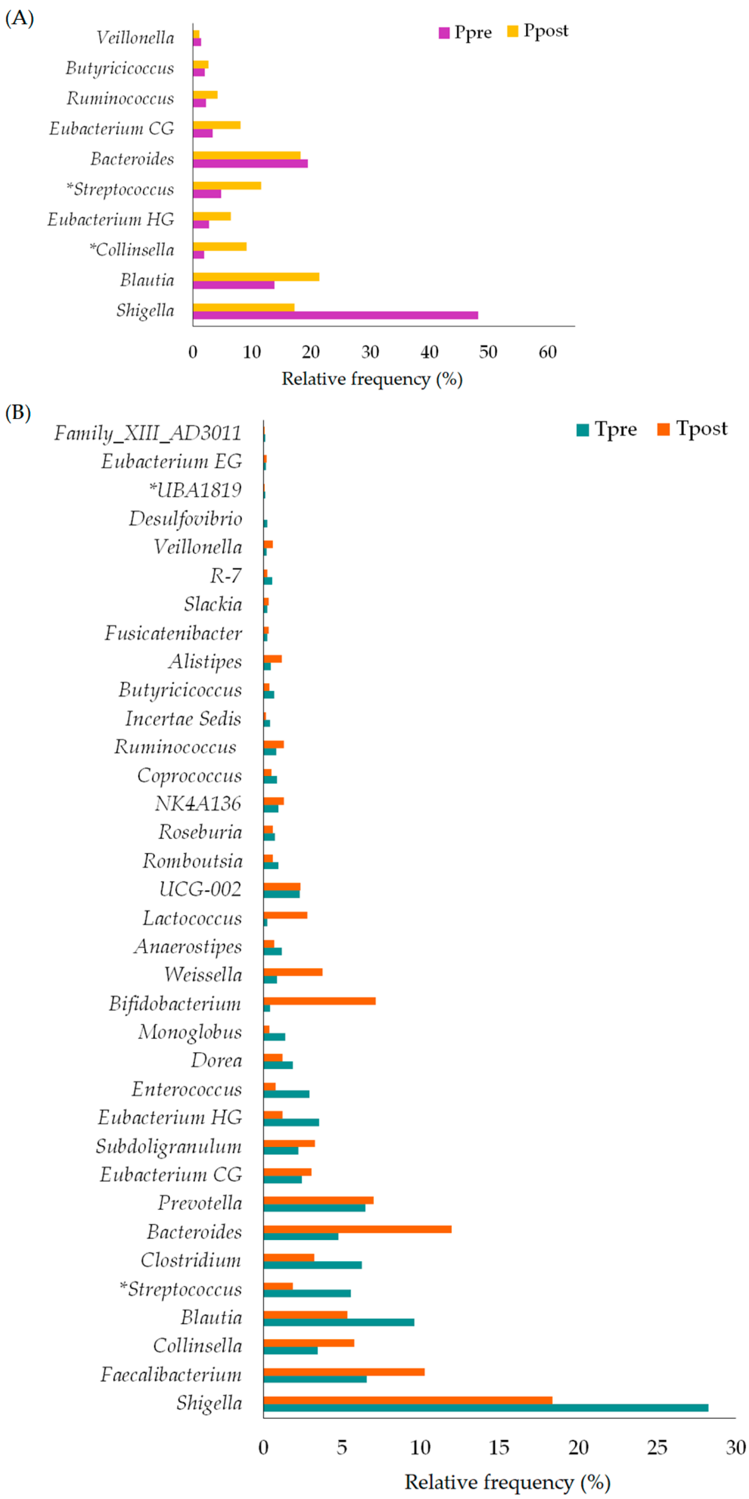

Figure S2. The relative frequency of estimated genera in the placebo (A) treatment (B) samples. Ppre: Placebo at baseline; Ppost: Placebo at week 12; Tpre: Treatment at baseline; Tpost: Treatment at week 12.

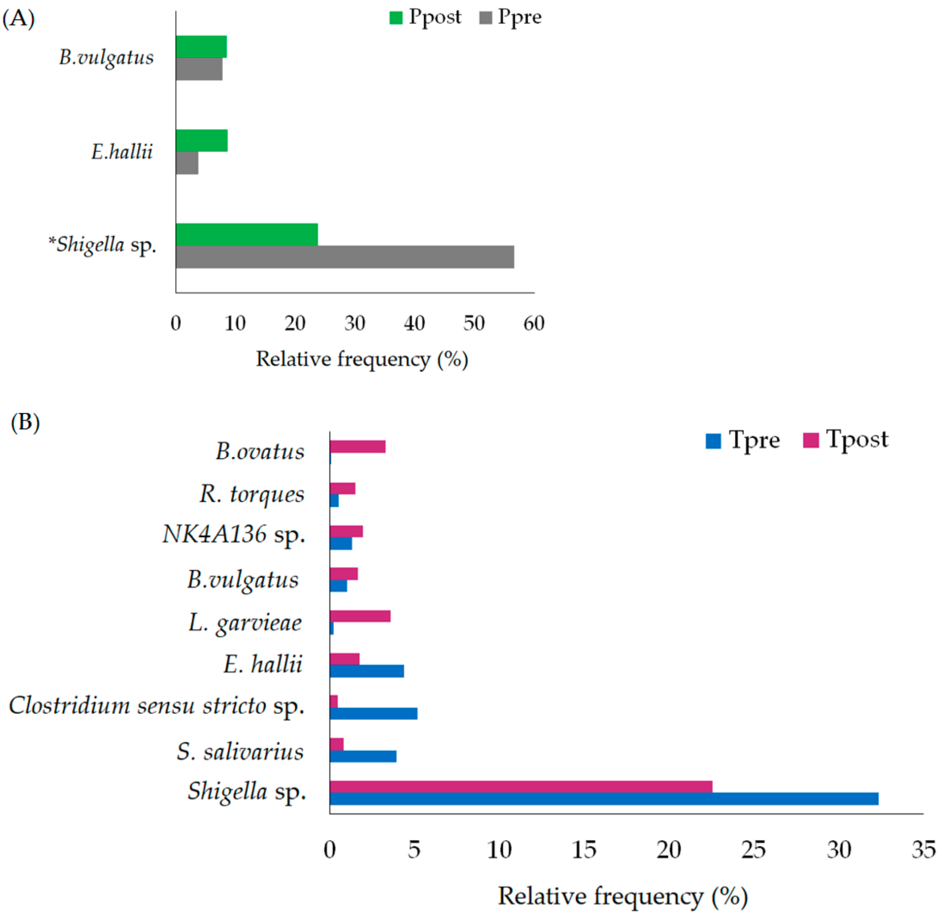

Figure S3. The relative frequency of estimated species in the placebo (A) treatment (B) samples. Ppre: Placebo at baseline; Ppost: Placebo at week 12; Tpre: Treatment at baseline; Tpost: Treatment at week 12.
